# Role of Phosphodiesterase 7 (PDE7) in T Cell Activity. Effects of Selective PDE7 Inhibitors and Dual PDE4/7 Inhibitors on T Cell Functions

**DOI:** 10.3390/ijms21176118

**Published:** 2020-08-25

**Authors:** Marianna Szczypka

**Affiliations:** Department of Pharmacology and Toxicology, Faculty of Veterinary Medicine, Wrocław University of Environmental and Life Sciences, Norwida 31, 50-375 Wrocław, Poland; marianna.szczypka@upwr.edu.pl; Tel.: +48-71-320-5215

**Keywords:** allergic and autoimmune diseases, intracellular cAMP, families of PDE, phosphodiesterase 7, PDE7, selective PDE inhibitors, T cells

## Abstract

Phosphodiesterase 7 (PDE7), a cAMP-specific PDE family, insensitive to rolipram, is present in many immune cells, including T lymphocytes. Two genes of PDE7 have been identified: PDE7A and PDE7B with three or four splice variants, respectively. Both PDE7A and PDE7B are expressed in T cells, and the predominant splice variant in these cells is PDE7A1. PDE7 is one of several PDE families that terminates biological functions of cAMP—a major regulating intracellular factor. However, the precise role of PDE7 in T cell activation and function is still ambiguous. Some authors reported its crucial role in T cell activation, while according to other studies PDE7 activity was not pivotal to T cells. Several studies showed that inhibition of PDE7 by its selective or dual PDE4/7 inhibitors suppresses T cell activity, and consequently T-mediated immune response. Taken together, it seems quite likely that simultaneous inhibition of PDE4 and PDE7 by dual PDE4/7 inhibitors or a combination of selective PDE4 and PDE7 remains the most interesting therapeutic target for the treatment of some immune-related disorders, such as autoimmune diseases, or selected respiratory diseases. An interesting direction of future studies could also be using a combination of selective PDE7 and PDE3 inhibitors.

## 1. Introduction

Phosphodiesterase (PDE) regulates intracellular levels of cyclic-3′,5′-adenosine monophosphate (cAMP) and cyclic-3′,5′-guanosine monophosphate (cGMP). It terminates their biological functions by converting them into inactive 5′-AMP and 5′-GMP, respectively. cAMP, as a second messenger, is an important factor regulating activity of many cells in the body, including immune cells. The intracellular level of cAMP depends on the activity of adenylyl cyclases (ACs), which are responsible for its synthesis, as well as on the activity of PDEs [1,2,3]. 

Phosphodiesterases are a superfamily of enzymes comprising at least 11 families (PDE1–PDE11), with numerous subtypes and isoforms [1,2,3]. These families are classified based on their molecular structure, sensitivity to exogenous and endogenous regulators, including PDE inhibitors, as well as their cellular and tissue location [1,2,3]. PDE families also differ in substrate affinity: PDE4, 7, and 8 specifically metabolize cAMP, PDE5, 6, and 9 preferentially hydrolyze cGMP, and PDE1, 2, 3, 10, and 11 inactivate both these cyclic nucleotides [1,2,3].

T cells, represented by heterogeneous populations of cells with diverse functions, play a crucial role in adaptive immune response. T cells are classified as CD4^+^ or CD8^+^ cells, according to the presence of CD4 or CD8 molecules on their surface. CD8^+^ T cells bind to peptides presented by the major histocompatibility complex (MHC) class I molecules, expressed on the surface of all nucleated cells. CD8^+^ T cells are cytotoxic T lymphocytes capable of directly destroying infected or malignant cells. CD4^+^ T cells recognize antigens presented by MHC class II molecules, expressed on the surface of antigen presenting cells (APCs), and they are mainly helper T cells (Th). A few subsets of helper T cells are distinguished: Th1, Th2, Th17, Th9, Th22, and T follicular helper cells (Tfh) [4,5]. Th1 cells enhance cell-mediated immune response mainly via production of IL-2 and IFN-γ; Th2 cells, which produce IL-4, IL-5, and IL-13, stimulate antibody secretion by B cells and thus enhance humoral immune response; Th17 cells exert pro-inflammatory activity mainly through the production of IL-17; Th9 and Th22 are defined by their high secretion of IL-9 and IL-22, respectively; and Tfh cells promote maturation and differentiation of B cells in the germinal center of follicles [5,6,7]. Regulatory T cells (Tregs), the large majority of which are CD4^+^, express a nuclear transcription factor, Forkhead box P3 (Foxp3), and are pivotal to maintaining peripheral immune tolerance and suppressing excessive immune response. For this reason, they play an important role in preventing autoimmune disorders, allergies, or transplant rejection [5,8].

In both CD4^+^ and CD8^+^ T lymphocytes, the predominant PDE families are PDE4 (in the soluble fraction) and PDE3 (in the particulate fraction), and the PDE family pattern in CD4^+^ and CD8^+^ cells is similar [9,10]. Additionally, PDE7 and PDE8, as well as a low activity of PDE5 were also detected in T lymphocytes [9,10,11]. PDE1 and PDE2 were reported as not detectable in T lymphocyte lysates [9], but another study demonstrated low activity of PDE1 and PDE2 in CD4^+^ and CD8^+^ T cell homogenates [10], as well as the presence of PDE11 protein in murine T cells [12].

To date, inhibition of the PDE4 family, as the main and best known PDE family in immune cells, was the principal strategy to increase intracellular cAMP level resulting in inhibition of inflammatory and immunological processes. For this reason, PDE4 inhibitors (especially rolipram, the first compound of this group) have been widely studied, and many research teams have demonstrated their inhibitory activity in inflammatory and immune cells, including T cells, and ensuing beneficial effects of these compounds in some autoimmune disorders or airway diseases [13,14,15]. Due to these properties, some selective PDE4 inhibitors are approved for the treatment of inflammatory or autoimmune diseases. Examples include roflumilast in chronic obstructive pulmonary disease (COPD) [16], apremilast in psoriatic arthritis and plaque psoriasis [17], and crisaborole for topical use in atopic dermatitis [18]. However, some side effects (mainly gastrointestinal), such as nausea, vomiting, diarrhea, or headache, are limitations of these therapies and may lead to treatment discontinuation [16,17], and new therapeutic solutions are still sought after. For this reason, some studies investigated whether inhibitors of other PDE families may affect T cell functions. They demonstrated that selective PDE3 or PDE5 inhibitors changed T cell subsets and activity [19,20,21,22,23]. 

Inhibition of the activity of the PDEs responsible for cAMP hydrolysis enhances cAMP signaling inside the cells. Among PDEs expressed in T lymphocytes, PDE7 and PDE8 are cAMP substrate specific PDEs, similarly to PDE4. Thus, inhibition of PDE7 seems an especially promising target for a therapeutic search with regard to regulation of T cell functions by modulating the cellular cAMP concentration.

## 2. cAMP Signaling in T Cells

The adenylyl cyclase family that catalyzes cAMP synthesis consists of ten isozymes: nine transmembrane ACs (tmACs, AC1–9) and one soluble AC (sAC, AC10) [24]. A membrane-bound isozyme AC7 is abundantly expressed in both T and B lymphocytes [25]. In T cells, cAMP synthesis through AC activation is induced by many different compounds, for example, adenosine, prostaglandins, or β-adrenergic agonists [26,27].

cAMP affects cell functions by activating both the canonical protein kinase A (PKA) pathway and non-canonical exchange protein activated by cAMP (EPAC) pathway [26]. PKA contains two regulatory and two catalytic subunits. Depending on the regulatory subunits, RI and RII, there are two types of PKA: PKA type I and PKA type II, respectively. There are also four distinct regulatory isoforms distinguished based on the presence of subunits α or β (RIα, RIβ, RIIα, and RIIβ). Among the four isoforms of the catalytic subunits, Cα and Cβ are the main ones, and Cγ and Cχ are less well characterized [26,28]. Once cAMP binds to the regulatory subunits of PKA, a conformational change occurs that leads to the release of catalytic subunits capable of phosphorylating the substrates [26,28]. PKA regulates several pathways by phosphorylation of a plethora of substrates, including those participating in T cell activation. Functionally, the most important PKA in T cells is PKA type I with regulatory subunit RIα [26,28]. 

In T cells, one of the most important substrates of PKA is *C*-terminal Src kinase (Csk). Csk activated by PKA phosphorylates lymphocyte-specific protein tyrosine kinase Lck, and this leads to the inhibition of Lck kinase activity. Thereby, the cAMP/PKA type 1/Csk pathway plays a key role in downregulation of T-cell receptor (TCR) signaling [26,27]. Other important PKA substrates in T cells are transcription factors, such as cAMP response element-binding protein (CREB), nuclear factor of activated T-cells (NFAT), and nuclear factor κB (NF-κB) [26,27].

PKA-mediated regulation involves A-kinase anchoring proteins (AKAPs) that bring PKA closer to the substrates, thus allowing for targeting and compartmentalization of PKA. However, AKAPs bind to various effectors and regulators of cAMP, not only PKA, but also ACs, PDEs, and other signaling molecules [26,27,29]. In T cells, seven AKAPs were detected: ezrin, AKAP79, AKAP149, AKAP450, AKAP95, AKAP220, and two AKAPs from the myeloid translocation gene (MTG) family: MTG8 and MTG16b [27]. Ezrin is an abundantly expressed AKAP in T cells responsible for PKA type I redistribution during immune synapse formation [26,27,29,30]. AKAPs differ in their ability to bind PDE4A and PDE7A: AKAP149, AKAP95, and MTG16b bind to PDE4A, whereas only MTG16b binds to PDE7A [29]. The role of AKAPs in the regulation of T cells was described in detail in a review by Wehbi and Taskén [27].

cAMP also initiates the non-canonical EPAC pathway. After binding cAMP, EPAC proteins (EPAC1 and EPAC2) activate Ras-associated proteins 1 and 2 (Rap1 and Rap2) that belong to the Ras family of small GTPases. In T cells, the main EPAC is EPAC1, for which the main target is Rap1. In T cells, Rap1 increases affinity of LFA-1 for ICAM-1, its ligand on antigen-presenting cells (APC), through evoking a conformational change of LFA-1. Hereby, Rap1 contributes to the formation of an immune synapse [26]. On the other hand, there are also reports that cAMP may inhibit Rap1 function in T cells [26].

A key factor for T cell activation is the appropriate spatiotemporal localization of proteins. T cells differentiate after their activation by APCs. This interaction is achieved by formation of an immune synapse that serves as a contact zone between a T cell and an APC constructed by central, peripheral, and distal supramolecular activation clusters (SMACs) [26,27]. Formation of SMACs is accompanied by accumulation of lipid rafts, membrane microdomains enriched in sphingolipids and cholesterol. They contain several important components involved in TCR signaling and act as signaling platforms. The molecules crucial for cell activation are transported near the immune synapse, while undesirable compounds are separated away to a distal pole complex (DPC) that is on an opposite side to the immune synapse. For example, after T cell activation, with participation of ezrin, PKA type I is transiently sequestered to the DPC, then, it is translocated near the immune synapse, and, finally, its distribution returns to that of the resting state [26,27,28,30]. Regarding PDEs, it is known that PDE4 is redistributed during T cell activation, after CD3/CD28 co-stimulation. Initially, PDE4 (especially PDE4B2) with β-arrestin is recruited to the lipid rafts near the immune synapse where PDE suppresses cAMP signaling in these membrane microdomains, and then it is accumulated in DPC [26,31]. This redistribution of components involved in cAMP signaling allows for creation of local cAMP pools inside the cells. The changes in cAMP signaling during formation of the immune synapse were described in detail in a recent review by Arumugham and Baldari [26].

The regulation of cAMP homeostasis differs between subtypes of T cells, for example, between regulatory T cells (Tregs) and other conventional T cells (Tcon). These differences include high expression of ACs and low expression of PDEs in Tregs, as compared with low expression of ACs and high expression of PDEs in Tcon [32]. Tregs accumulate cAMP and then suppress Tcon responses by direct cAMP influx through gap junctions to target cells or by inducing adenosine action that leads to stimulation of adenylyl cyclases in Tcon. The cAMP intracellular pathway in Treg suppression was described in detail in a review by Rueda et al. [32].

## 3. Phosphodiesterase 7 (PDE7) Family in T Cells

PDE7, a cAMP-specific PDE insensitive to rolipram, was firstly isolated by Michaeli et al. in 1993 at the gene level from a human glioblastoma cDNA library as a new cAMP-specific PDE [33]. In the same year, Ichimura and Kase [34] described the presence of cAMP-specific PDE insensitive to rolipram in a Jurkat T cell line. Two genes of PDE7 were identified: PDE7A and PDE7B [35,36,37]. PDE7A has three splice variants, PDE7A1, PDE7A2, and PDE7A3 [1,35,38]. PDE7A1 occurs in both cytosolic and particulate fraction, while PDE7A2 is located only in particulate cellular fraction [1,35]. Four splice variants of PDE7B (PDE7B1, PDE7B2, PDE7B3, and a variant found in GenBank) were identified [2,3,39]. 

The presence of PDE7 in the soluble fraction of T cells, both CD4^+^ and CD8^+^, was detected by Giembycz et al. [9]. The predominantly expressed splice variant of PDE7 in human lymphoid tissues is PDE7A1 [40], and it is distributed ubiquitously in many types of human proinflammatory and immune cells, including T lymphocytes, both CD4^+^ and CD8^+^, as well as in T cell lines Jurkat and HUT-78 [38,41,42]. Nueda et al. [43] reported that PDE7A1 mRNA was expressed much more abundantly in human resting peripheral naïve T cells (CD4^+^CD45RA^+^ cells) than in memory T cells (CD4^+^CD45RO^+^ cells). PDE7A1 is an enzyme with bifunctional properties, it suppresses cAMP signaling either via cAMP hydrolysis or by binding to the catalytic subunit of PKA causing direct inhibition of PKA activity [2,44]. A study conducted by Asirvatham et al. [29] on T-cell lines (Jurkat cells) demonstrated that in T cells, PDE7A was located in the Golgi apparatus. 

Apart from PDE7A1, HUT-78, a human T cell line, expresses also PDE7A3 [11,38]. mRNA for PDE7A2 was detected in immune cells, including peripheral blood T lymphocytes and T cell lines, but the enzyme protein was not detected [41]. 

The expression of PDE7B was confirmed by Zhang et al. [45] in peripheral blood mononuclear cells (PBMC) and normal B cells from healthy adults. However, they demonstrated higher levels of PDE7B mRNA and protein expression in B leukemic cells than that in PBMC from healthy adults [45]. On the other hand, the expression of PDE7B in T leukemic cell lines was extremely low [46].

Jones et al. [47] reported similar expression of both PDE7A and PDE7B mRNA in human CD4^+^ cells from healthy and asthmatic donors as well as in human CD8^+^ cells from healthy, asthmatic, and COPD donors. Interestingly, no difference in PDE7A and PDE7B expression was demonstrated between healthy patients and patients with respiratory diseases [47].

Studies on the relationship between T cell activation and PDE7 expression or activity demonstrated that PDE7 may be essential for T cell activation. Li et al. [40] showed PDE7A1 mRNA expression in resting T cells. Co-stimulation of CD3 and CD28 receptors on T cells led to an increase in PDE7 activity and in consequence to a decrease in the cAMP intracellular level, a rise in IL-2 level and stimulation of T cell proliferation [40]. This effect was prevented by using of PDE7 antisense oligonucleotide. Likewise, Glavas et al. [11] reported that in human CD4^+^ T cells both PDE7A1 and PDE7A3 were present and they were upregulated during CD3/CD28 stimulation. Consistent with these findings, Kanda and Watanabe [48] also demonstrated that the expression of PDE7 mRNA in human resting T cells was increased by CD3/CD28 stimulation, but not by phytohemagglutinin (PHA). They concluded that PDE7 expression may depend on the type of the stimulus. These results are at odds with the study of Nueda et al. [43], who reported that both in naïve and memory T cells PDE7A1 mRNA was not upregulated during CD3/CD28 co-stimulation. Nakata et al. [49] compared the effects of two PDE inhibitors on human PBMC activity: T-2585, a high potency inhibitor of PDE4 (IC_50_ = 0.00013 µM) and a low potency inhibitor of PDE7A (IC_50_ = 1.7 µM), and RP 73401 (piclamilast), a potent selective PDE4 inhibitor (IC_50_ = 0.00031 µM). T-2585 suppressed IL-5 production, proliferation of PBMC, expression of IL-2, IL-4, and IL-5 mRNA, and expression of CD25 on T cells, while RP 73401 triggered only weak inhibition of these parameters. Based on these results the authors suggested that PDE7 may play a crucial and PDE4 a supplemental role in T cell activity [49].

According to other studies, the activity of PDE7 is not crucial for T cell activation. Yang et al. [50], in a study using knockout PDE7A-deficient mice, demonstrated that PDE7A is not indispensable for T cell activation. No inhibition in T lymphocyte proliferation or cytokine production by Th1 and Th2 cells after CD3/CD28 co-stimulation was observed. A similar conclusion resulted from a study conducted by Chevalier et al. [51], who reported that PDE7 was not involved in an experimental model of asthma in ovalbumin-immunized mice. A deletion of the PDE7B gene in knockout mice had no effect on the following tested parameters: airway hyperreactivity, cytokine levels in bronchoalveolar lavage, inflammatory cell infiltration, and total IgE in serum. In the same study, a similar lack of effects on the tested parameters was observed after pharmacological inhibition of PD7A and PDE7B. Kanda and Watanabe [48] reported that PDE7 antisense oligonucleotide only slightly inhibited the production of IL-13 by anti-CD3/CD28-stimulated T cells, and they also concluded that the role of PDE7 was not pivotal. 

The appearance of selective PDE7 inhibitors allows for further clarification of the PDE7 role in T cell activity.

## 4. Potential Therapeutic Use of Selective PDE7 Inhibitors 

PDE7 inhibitors belong to different chemical groups, with wide structural diversity, for example, thiadiazole derivatives [52], sulfonamide derivatives [42], thioxoquinazoline derivatives [53], pyrimidine-based inhibitors [54], furan derivatives [55], or barbituric acid derivatives [56].

To date, the main research goal for PDE7 inhibitors is to determine their effects with regards to neurodegenerative diseases, respiratory disorders, and some cancer diseases (Table 1). 

Benefits of PDE7 inhibitors were reported in the treatment of experimental autoimmune encephalomyelitis (EAE) in mice, as an animal model of human multiple sclerosis [52,55,57,58,59,60], spinal cord injury [61], a murine model of Alzheimer’s disease [62,63], a rodent model of Parkinson’s disease [64,65], as well as in sevoflurane-induced long-term memory deficits in mice [66].

Regarding respiratory diseases, PDE7 inhibitors may have beneficial effects in asthma and COPD due to relaxation of airway smooth muscles and anti-inflammatory properties [67,68], as well as in smoke-induced lung inflammation in mice [69]. However, Chevalier et al. [51] showed no effects of PDE7 inhibition in an experimental model of asthma in mice.

Some studies demonstrated that PDE7 inhibitors, PDE4/7 inhibitor or a combination of PDE3, 4, and 7 inhibitors induced apoptosis of leukemic cells [45,46,70]. It has also been shown that selective PDE7 inhibitors inhibited breast cancer cell migration [71].

For a review on advances in chemical and biological research concerning selective and dual inhibitors of PDE7, the readers are referred to a recent review by Jankowska et al. [72].

## 5. The Effect of Selective PDE7 Inhibitors on T Cell Activity

In many applications mentioned above, the anti-inflammatory and immunosuppressive properties of PDE7 inhibitors, resulting from their suppressive effects on immune cells, are beneficial. Therefore, studies that focus on the effects of these compounds on T cell functions allow us to broaden our understanding of the PDE7 role.

Smith et al. [42] reported an inhibitory effect of BRL-50481, an inhibitor of PDE7A1 (IC_50_ = 0.26 and 2.4 µM, at 0.05 µM and 1 µM of cAMP, respectively) on T cells, provided that PDE4 was inhibited simultaneously. Although the content of cAMP in MOLT-4 T cells increased in the presence of BRL-50481, even when the compound was used alone, the inhibitor did not affect proliferation of CD8^+^ T cells induced by IL-15. Interestingly, it potentiated the inhibitory effect of rolipram. This additive inhibitory effect of the PDE7 inhibitor and rolipram was confirmed by Jones at al. [47]. However, in that study, PF 0332040, another PDE7 inhibitor (IC_50_ for PDE7A1 = 0.088 µM, IC_50_ for PDE7B = 0.76 µM), even when used alone, reduced PHA-stimulated proliferation of human peripheral blood mixed mononuclear cells, in a manner similar to that of rolipram [47].

In a study conducted by Nueda et al. [43], two PDE7A inhibitors, LASW404 and LASW405 (IC_50_ = 0.78 and 0.011 µM, respectively), suppressed the production of IL-2 and proliferation of CD3/CD28-stimulated naïve and memory T cells but only at high concentrations of 1 µM and 10 µM (not below these levels). Moreover, LASW404 did not boost cAMP level in these cells, while LASW405 caused only a slight increase in cAMP concentration. The authors concluded that the inhibition of T cells function did not result directly from blockage of PDE7A1 activity. Consistent with that study, Guo et al. [54] also suggested that the inhibitory impact on T cells was unrelated to PDE7 inhibition. They synthesized pyrimidine-based PDE7 inhibitors that successfully reduced T cell proliferation. However, these inhibitors had the same effect on splenocytes derived from wild type mice and PDE7 knockout mice [54]. 

Kadoshima-Yamaoka et al. [73] showed that ASB16165, a PDE7A inhibitor (IC_50_ = 15 nM for human PDE7A, IC_50_ for PDE4 = 2.1 µM), reduced IL-12-induced IFN-γ synthesis by murine T lymphoblasts stimulated by the anti-CD3 antibody in a cAMP/PKA-dependent manner. ASB16165 also decreased CD3/CD28-stimulated T cell proliferation and the production of IL-2 and IFN-γ by these cells. Interestingly, the inhibitory effect of ASB16165 was similar to that of rolipram, a selective PDE4 inhibitor. The same authors reported in another study [74] an ASB16165-mediated reduction in the formation and cytolytic activity of CD8^+^ cells (cytotoxic lymphocytes), induced by a mixed lymphocyte reaction (MLR). The compound also inhibited development and proliferation of CD8^+^ and CD4^+^ T cells in MLR, and the effects of ASB16165 on CD8^+^ cells were stronger than the effects of rolipram. These results suggested that PDE7, not PDE4, was crucial to the generation and activity of cytotoxic T cells [74].

Dong et al. [46] detected PDE3B, PDE4A, PDE4D, PDE7A, and PDE8A in T leukemic cell lines. PDE7 mRNA was found in all tested cell lines, CEM (CEM-S2 and CEM-R8) and Jurkat, while PDE7 protein expression was detected only in CEM lines. In that study, selective PDE3, 4, or 7 inhibitors used alone had a slight effect on cell viability. However, a combination of all three inhibitors significantly enhanced glucocorticoid-induced apoptosis of T leukemic cells [46]. 

Using EAE mice, González-García et al. [57] examined the effects of two PDE7 inhibitors, TC3.6 and BRL-50481, vs. the PDE4 inhibitor rolipram on the course of EAE and T cell activity. They found that the efficacy of TC3.6 in preventing EAE was similar to that of rolipram. Both TC3.6 and rolipram reduced cell infiltration in the central nervous system (CNS) as well as inhibited T cell proliferation, increased the expression of Foxp3 (an intracellular marker of regulatory T cells), and decreased IL-17 levels. However, the reduction of IL-17 synthesis by TC3.6 and rolipram resulted from distinct pathways. TC3.6 exerted a direct inhibitory effect on CD4^+^ cells, while rolipram decreased IL-17 synthesis indirectly through the expansion of Tregs and stimulation of the production of IL-27, an inhibitor of Th17 cells. Interestingly, BRL50481 had no effect on the course of the disease, probably due to its physico-chemical properties, for example, low cell penetrability [57].

Martín-Álvarez et al. [58] also examined the effects of PDE7 inhibitors in mice with EAE. They reported that VP3.15 (IC_50_ = 1.59 µM) reduced the clinical symptoms of EAE (at a dose of 10 mg/kg, intraperitoneally), similar to the action of fingolimod, a drug used in the treatment of multiple sclerosis, and was more effective than another PDE7 inhibitor, BRL-50481. Either of the two PDE7 inhibitors ameliorated neuropathological scores in the spinal cord. In the study conducted in vitro, VP3.15 inhibited splenocyte proliferation and TNF-α production by these cells. An increase in PDE4B2 mRNA expression in the spinal cord was observed in BRL-50481-treated mice (but not in VP3.15-treated animals), probably as a compensatory reaction [58].

Xu et al. [56] identified barbituric acid derivatives as PDE7 inhibitors, and one of them, BC12 (IC_50_ = 0.77 µM), exhibited a potent immunosuppressive effect. BC12 inhibited IL-2 production in the culture of Jurkat T cells stimulated with a combination of phorbol-12-myristate-13-acetate (PMA) and PHA. This effect was stronger than that of rolipram or BC54, a dual PDE4/7 inhibitor, and more potent towards PDE7 than was BC12. BC12-4, an analog of BC12 that did not show PDE7 inhibitory activity, exerted a similar effect. These findings showed that BC12 reduced IL-2 production not through a PDE7-dependent mechanism, but via prevention of many gene expressions in stimulated Jurkat T cells, including IL-2 mRNA. The diminution in IL-2 production by BC12 was also observed in a study with primary human and murine T cells stimulated by anit-CD3/anti-CD28 antibodies or by PMA plus ionomycin, respectively. Additionally, both BC12 and BC12-4 inhibited T cell proliferation of murine T splenocytes [56].

Regarding Tregs, González-García et al. [57] demonstrated that PDE7 and PDE4 inhibition increased the expression of the Treg marker, Foxp3. These data are consistent with the results of other studies [22,75,76,77] that showed that PDE inhibition leads to an increase in subsets of Tregs and enhances their inhibitory effects on other Tcon.

## 6. The Effect of Dual PDE4/7 Inhibitors on T Cell Activity

The use of selective PDE4 inhibitors in treatment is complicated or restricted due to their common side effects. For this reason, another strategy has been developed, in other words, searching for less selective compounds that inhibit more than one cAMP-specific PDE family.

Several previous studies mentioned above [42,46,47,49,67] indicated that simultaneous inhibition of PDE4 and PDE7 provides a synergistic inhibitory effect on T lymphocyte activity, which can be used to weaken T cells in the therapy of diseases involving their hyperactivity. Fortin et al. [69] also reported that the anti-inflammatory effects of PDE4 inhibitors may be improved by additional blocking of PDE7. In that study, inhaled antisense oligonucleotides against PDE4B/4D and 7A protected lungs against cigarette smoke-induced inflammation in mice. Regarding B cells, Zhang et al. [45] demonstrated that not only BRL-50481 and IR-202, inhibitors of PDE7, but also IR-284, a dual PDE4/PDE7 inhibitor, increased apoptosis in chronic lymphocytic leukemia (CLL) cells. Castaño et al. [53] showed that PDE7 inhibitors and dual PDE4/7 inhibitors reduced T cell viability (murine T-cell line D10.G4.1). Therefore, one of the therapeutic strategies involves dual PDE4/7 inhibitors, and compounds expressing this double activity are still being sought for the treatment of immune-related disorders [78,79]. 

Some studies concerning dual PDE4/7 inhibitors examined their effects on T cells. Yamamoto et al. [80,81,82] investigated the properties of YM-393059, a dual PDE7A and PDE4 inhibitor (IC_50_ for PDE7A = 14 nM and IC_50_ for PDE4 = 630 nM). They found that YM-393059 inhibited both Th1 (IL-2, IFN-γ) and Th2 (IL-4) cytokine synthesis by murine splenocytes in vitro. However, this effect was weaker than the effect of a selective PDE4 inhibitor, YM976. Both compounds had similar inhibitory effects on anti-CD3-induced IL-2 production after oral administration in mice. The results of that study also suggested fewer side effects of the tested PDE7A/4 inhibitor as compared with those from a PDE4 inhibitor [82]. YM-393059 was effective both in acute and chronic inflammation models in mice (but not in rats with the carrageenan-induced edema model) [81], as well as in a collagen-induced arthritis model in mice [80]. YM-393059 reduced serum IgE and IgG levels in chronic inflammation models and a collagen-induced arthritis model in mice, respectively [80,81], but it did not prevent acute rejection of cardiac allografts in rats [80].

De Medeiros et al. [70] demonstrated that BC54, an inhibitor of PDE4 and PDE7 (IC_50_ = from 50–110 nM for PDE4A, PDE4B, PDE4D; IC_50_ = 140 nM for PDE7A and PDE7B) exhibited anti-inflammatory properties (reduction of TNF-α production by macrophages and decrease in IL-2 synthesis by PMA-PHA-stimulated Jurkat T cells) and elicited apoptosis in CLL cells. Furthermore, this action outperformed the effect of a combination of rolipram and BRL-50481 [70].

Taking into consideration the synergistic effects of PDE4 and PDE3 inhibitors and beneficial effects of dual PDE3/4 inhibitors in the treatment of respiratory diseases [83,84,85], it seems likely that simultaneous inhibition of the PDE7 and PDE3 families could also be a promising therapeutic target.

## 7. Concluding Remarks 

Sustained high intracellular levels of cAMP suppress T cell functions. However, it is known that the regulatory effect of cAMP on T lymphocytes is dual in nature. A transient cAMP rise after T-cell receptor (TCR) ligation is required for T cell activation [26,86]. It is hypothesized that these opposite effects may be partially explained by cAMP spatial and temporal compartmentalization at the subcellular level, in which PDE activity plays an essential role. PDE-mediated cAMP compartmentalization is believed to result from redistribution of PDE to the sites of cAMP formation, and it allows the body to regulate local concentrations of cAMP inside the cell [26].

It is known that PDE4 is redistributed during immune synapse formation [26,31]. There is no information about redistribution of PDE7 during T cell activation. Further studies are required to reveal the role of PDE7 in cAMP compartmentalization. Based on the available results, PDE7 is located in the Golgi apparatus and interacts with AKAP proteins, but in a different manner than does PDE4 [29], which may be the reason for some differences between the effects of PDE4 and PDE7.

Activity of the intracellular cAMP-pathway elements differs between subtypes of T cells, especially in Tregs where lower PDE expression was found as compared with that from other Tcons [32]. PDE inhibition may alter the activity of T cells not only by direct inhibitory action but also through an increase of the suppressing role of Tregs. Moreover, there are differences in response to changes of cAMP levels between CD8^+^ and CD4^+^ cells, as well as between Th subsets. Cytotoxic lymphocytes (CD8^+^ cells), unlike CD4^+^ cells, were found to be more sensitive to PDE7 than PDE4 inhibition [74]. Th1 cell activity was inhibited to a greater extent by cAMP-elevating agents than Th2 cell activity [87]. In addition, cAMP may alter the balance between Th1, Th2, and Th17 cells towards Th17 cells [88,89]. Regarding Th1 and Th2 subsets, the data are ambiguous; some authors indicated that elevation of cAMP promoted Th1 differentiation [89], while other studies showed an opposite effect: Th2 cell development [90]. A balance between different T cell subsets is required for proper functioning of the immune system. A disruption of the Th1/Th2 balance leads to immune dysfunctions such as allergic disorders in the case of predominant Th2 cell activity or autoimmune diseases when the activity of Th1 cells prevails [6,8].

Additionally, this situation is complicated by the fact that some studies indicate the existence of compensatory reactions. For example, an increase in the expression of PDE4 was observed when using a PDE7 inhibitor [58]. The existence of compensatory mechanisms may be also indicated by the fact that a lack of PDE7 did not interrupt T cell function [50]. Moreover, the results of other studies suggested that PDE7 inhibitors affected T cells through a PDE7-independent mechanism [43,54,56].

Taken together, the role of PDE7 inhibitors in T cell activity is still unclear and answers to many questions are required. Based on the results discussed, the effects of selective PDE7 inhibitors on T cell functions are ambiguous. In most research studies using PDE7 inhibitors, PDE7 blockage suppressed T cell activity. However, this effect is more unequivocal when PDE4 is inhibited at the same time. Considering the synergistic effect of PDE4 and PDE7 inhibition on T cell activity, it seems that dual PDE4/7 inhibitors or a combination of selective PDE4 and PDE7 inhibitors is the most promising therapeutic strategy to inhibit or modulate T cell functions through an increase in intracellular cAMP concentration. Another interesting area would be investigating the effects of a combination of selective PDE7 and PDE3 inhibitors. 

## Figures and Tables

**Table 1:** Potential therapeutic use of selective phosphodiesterase 7 (PDE7) inhibitors.

PDE7 Inhibitors	Disease Model/Disease	References
5-imino-1,2,4-thiadiazole derivative: “compound 15”	experimental autoimmune encephalomyelitis (EAE)	Redondo et al., 2012 [52]
furan derivative: “derivative 13”		Redondo et al., 2012 [55]
TC3.6 (BRL-50481: no effect)		González-García et al., 2013 [57]
VP3.15, BRL-50481		Martín-Álvarez et al., 2017 [58]
VP3.15		Medina-Rodríguez et al., 2017 [59]
TC3.6		Mestre et al., 2015 [60]
S14, VP1.15	spinal cord injury	Paterniti et al., 2011 [61]
S14 S14	murine model of Alzheimer’s disease	Bartolome et al., 2018 [62] Perez-Gonzalez et al., 2013 [63]
S14	rodent model of Parkinson’s disease	Morales-Garcia et al., 2015, 2020 [64,65]
BRL-50481	sevoflurane-induced long-term memory deficits in mice	Chen et. al., 2020 [66]
BRL-50481	respiratory diseases: asthma, COPD	Mokry et al., 2013 [67] Page, 2014 [68]
“compound 21a”: no effect	induction of apoptosis of leukemic cells	Chevalier et al., [51]
BRL-50481 (in combination with rolipram)		de Medeiros et al., 2017 [70]
Spiroquinazolinone		Dong et. al, 2010 [46]
BRL-50481, IR-202		Zhang et al., 2008 [45]
Spiroquinazolinone	inhibition of breast cancer cell migration	Dong et al., 2015 [71]

## Abbreviations

AC	Adenylyl cyclase
AKAP	A-kinase anchoring protein
APC	Antigen presenting cell
cAMP	Cyclic-3′,5′-adenosine monophosphate
CD	Cluster of differentiation
cGMP	Cyclic-3′,5′-guanosine monophosphate
CLL	Chronic lymphocytic leukemia
COPD	Chronic obstructive pulmonary disease
CREB	cAMP response element-binding protein
Csk	C-terminal Src kinase
DPC	Distal pole complex
EAE	Experimental autoimmune encephalomyelitis
EPAC	Exchange protein activated by cAMP
Foxp3	Forkhead box P3
IC	Inhibitory concentration
MHC	Major histocompatibility complex
MLR	Mixed lymphocyte reaction
MTG	Myeloid translocation gene
NFAT	Nuclear factor of activated T-cells
NF-κB	Nuclear factor κB
PBMC	Peripheral blood mononuclear cells
PDE	Phosphodiesterase
PHA	Phytohemagglutinin
PKA	Protein kinase A
PMA	Phorbol-12-myristate-13-acetate
sAC	Soluble adenylyl cyclase
SMAC	Supramolecular activation cluster
Tcon	Conventional T cell
tmAC	Transmembrane adenylyl cyclase
TCR	T-cell receptor
Treg	Regulatory T cell
